# Grade prediction in the middle east: a post-pandemic case study of the optimism bias

**DOI:** 10.3389/fpsyg.2023.1270621

**Published:** 2024-01-23

**Authors:** Maura A. E. Pilotti, Khadija El Alaoui, Arifi Waked

**Affiliations:** ^1^Department of Sciences and Human Studies, Prince Mohammad Bin Fahd University, Khobar, Saudi Arabia; ^2^Cognitive Science Center, Prince Mohammad Bin Fahd University, Khobar, Saudi Arabia

**Keywords:** grade prediction, accuracy, confidence, middle east, illusion of knowing, optimism bias

## Abstract

Evidence exists that the pandemic has brought about stress, and altered study habits and academic performance. No evidence exists regarding whether metacognition has also been altered. The present field study examined the accuracy and confidence with which college students make grade predictions in a general education course after the pandemic. It tested whether one of three types of biases affected students’ predictions as a way to cope with the uncertainty of a final exam’s outcome: illusion-of-knowing, optimism, and pessimistic bracing. Students made predictions both before and after completing the final exam (summative assessment) to determine the impact of each of the hypothesized biases on estimates made in a context of varying uncertainty. Accuracy was computed as the difference between expected and actual grades on the final exam. Confidence in the predictions made was measured on a Likert scale. Exam performance was categorized as good, poor, or inadequate. In this study, less-than-desirable performance was accompanied by overestimations. However, overestimations were made with little confidence and benefited from the information acquired from completing the exam. This pattern of results suggests that students who are not doing well are not under the spell of the illusion-of-knowing phenomenon. Indeed, their optimistic predictions are punctured by the awareness of a likely undesirable outcome (as indicated by their weak confidence in the predictions made). Implications and applications of these findings are discussed.

## 1 Introduction

In educational settings, the illusion-of-knowing bias refers to students who mistakenly believe that they have mastered materials and/or skills (Avhustiuk et al., 2018; Pilotti et al., 2019; Torres et al., 2023). Blindness to the discrepancy between perceived and actual knowledge has two correlates: lack of awareness of one’s deficiencies and preserved confidence in one’s abilities and knowledge to carry out the task at hand. Its measurement entails asking students to predict their performance, usually in the form of a grade on a test or assignment, or as a course grade at the end of the semester. If learners are aware of their current knowledge and skills, they will be rather accurate in their estimated grades. Overestimations imply learners’ erroneous presumption that they know the information and skills demanded by a particular task.

Several studies have shown that the illusion-of-knowing bias is likely to be possessed by students who exhibit poor performance (Pilotti et al., 2019, 2021). Students who do well are usually reasonably aware of what they know and do not know, thereby yielding rather accurate or even pessimistic estimates (Dunning et al., 2003; de Carvalho Filho, 2009; Paik and Schraw, 2013).

The illusion-of-knowing bias implies that students are not only oblivious to their lack of knowledge and skills but also confident of owning the skills and knowledge they do not have. Studies that have examined both awareness and confidence have questioned the bias (Miller and Geraci, 2011a; Al Kuhayli et al., 2019; Hamann et al., 2020). These studies have reported that when students with poor or deficient performance make optimistic estimates regarding academic outcomes, they do so with much less confidence than that expressed by good performers. Thus, poor or deficient performers are not “blissfully incompetent” (Williams, 2004), “unskilled” or “unaware” (Ehrlinger et al., 2008; Serra and DeMarree, 2016) as the illusion-of-knowing phenomenon would imply.

The lack of confidence of poor and deficient performers (Pilotti et al., 2021) suggests these students are under the spell of a different bias: optimism (Sharot, 2011; Lefebvre et al., 2017). Under such a bias, students predict a more desirable outcome to better cope with a distressing and disappointing outcome that is viewed as inevitable. Their overestimations are a form of wishful thinking that temporarily safeguards their self-concept from the outcome they anticipate. Thus, when the anticipated outcome is undesirable, metacognitive monitoring (Gutierrez et al., 2016), which entails attention to cognitive activities to foster regulation, operates on two fronts, each with its distinct motives. The affective front seeks to preserve one’s self-image through optimistic predictions. The cognitive front, instead, seeks to maintain one’s grip on reality. Low confidence in the predictions made is a tacit recognition that such reality cannot be escaped.

Of course, there is always the possibility of a pessimistic-bracing bias. Indeed, evidence exists that under conditions of uncertainty, individuals brace for potentially negative outcomes by lowering their expectations (Sweeny et al., 2006; Sweeny and Andrews, 2014). Sweeny and colleagues called the phenomenon “adaptive pessimism” because it represents a strategy that enhances an individual’s ability to cope with the distress and disappointment associated with an upcoming negative outcome. The phenomenon has been shown in many settings (Sweeny, 2018). For instance, people who are awaiting the results of a cancer screening test predict the worst outcome. Students lower their expectations about their exam performance to brace for the worst and minimize the blow of bad news (Sweeny and Shepperd, 2010; Sweeny et al., 2016). In such a case, students will underestimate their likely performance and be reasonably confident in the undesirable outcome they anticipate.

When would grade prediction be shaped by pessimism rather than optimism? Evidence suggests that the pessimistic-bracing bias is likely to be engaged when students perceive the communication of undesirable outcomes as temporally close. According to Sweeny et al. (2006), distant outcomes tend to be construed more abstractly than near outcomes. Thus, people’s desires are more likely to contaminate people’s predictions about distant events, leading to optimistic predictions, than those about near events for which attention on what is likely to happen is unescapable (Trope and Liberman, 2003). For instance, at the beginning of the semester, students may predict that they will do well in all their courses. Even though opportunities for controlling outcomes dissolve after the final exams in challenging courses (Shepperd et al., 1996), outcomes will not be known until sometime later. Thus, optimism may persist. But, as the day of disclosure approaches, Sweeny et al. (2006) expect students’ estimates to shift from optimistic to pessimistic.

Understanding the source of students’ erroneous predictions is important. The judgments that result from erroneous metacognitive monitoring are consequential sources of students’ regulation of cognitive activities during learning, thereby defining the suitability of corrective interventions (Zabrucky, 2010). Indeed, if students overestimate their abilities and current level of knowledge when dealing with the acquisition and retention of instructional material, they will not devote enough attention and effort to learning it. They will mistakenly believe that they have already mastered the material or that not much effort will need to be exerted to master it. Instead, if students underestimate their abilities and current knowledge, they will expend unnecessary effort and squander valuable time processing information already learned or practicing skills that have been already mastered.

Understanding the sources of students’ erroneous predictions relies on an examination of the underlying cognitions. Such cognitions not only shape students’ behaviors associated with learning activities but also determine the nature of the interventions that can satisfactorily address students’ counterproductive behaviors. Indeed, if students’ meager efforts reflect unawareness of personal shortcomings, corrective measures may be different from those needed for students whose meager efforts reflect their hopelessness or wishful thinking (Hacker et al., 2000; Miller and Geraci, 2011b; Saenz et al., 2019).

A critical aspect of the task of understanding the cognitions underlying students’ erroneous predictions is the context within which such predictions are made. The present study focuses on the aftermath of the COVID-19 pandemic since little evidence exists on the matter. To this end, it is noteworthy to point out that a disruptive event, such as the pandemic, is one capable of altering lives (Mutch, 2014; Burchard, 2019). Not surprisingly, social isolation and disrupted social interactions resulting from the pandemic have been linked to changes in brain functioning (Bzdok and Dunbar, 2022). Increased stress has been associated with the uncertainties that altered people’s established routines and the helplessness that resulted from the loss of significant others (Panther et al., 2021). Stress may impair learning and memory in different ways (Vogel and Schwabe, 2016). Even though stress may boost memory formation, thereby leading to lasting memories, stress can impair memory retrieval, potentially depressing performance. Stress may also disrupt the integration of new information into memory records (i.e., updating) as well as deprive learning processes of their flexibility by promoting rigid, habit-like actions. Excessive apprehension, a likely symptom of stress, can impair decision-making (Porcelli and Delgado, 2017; da Silva Castanheira et al., 2021) through its impact on the functioning of working memory. Working memory is a system that allows learners to maintain and manipulate information in their minds for several seconds to plan, make decisions, and execute a variety of tasks. Worrying disrupts executive functioning, slows the speed of information processing, and depletes working memory of cognitive resources, thereby reducing the amount of information available to learners for task execution (Beckwé et al., 2014; da Silva Castanheira et al., 2021). Most troubling is that deficiencies in the functioning of working memory may become more severe over time and be linked to the incidence of fatigue (Trezise and Reeve, 2016).

Evidence that the pandemic may have changed brain functioning (Bzdok and Dunbar, 2022) begs the question of whether the pre-pandemic patterns reported by several researchers (e.g., Miller and Geraci, 2011a; Al Kuhayli et al., 2019; Hamann et al., 2020) would be replicated in the aftermath of such a disruptive event. The task of adequately estimating performance on an exam requires that students consider the accessibility of key exam materials in their mind against likely test demands, take into account the uncertainties of the information available, and then generate predictions. In other words, the task requires students to retain complex and uncertain information in working memory to estimate performance that is yet to materialize. Evidence exists that decision-making is disrupted by stress. In contexts of uncertainty, stress affects executive functions that heavily rely on working memory (Morgado et al., 2015). Such effects entail an over-reliance on automatic response tendencies accompanied by declines in controlled cognitive processes because stress weakens the resources needed by executive functions to make adjustments (Starcke and Brand, 2012). Endogenous stress is also likely to add to stress arising from common academic circumstances (e.g., an exam). As such, stress can be detrimental to students’ ability to cope with situations that require reliance on controlled cognitive processes, such as the self-regulation processes involved in metacognition. Disruptions also affect the learner’s evaluation of the valence of the anticipated outcomes. The evidence is unclear though as to the nature of the impact (Porcelli and Delgado, 2017). Stress has been found to increase the salience of desirable outcomes (Mather and Lighthall, 2012), or blunt learners’ sensitivity to such outcomes (Bogdan and Pizzagalli, 2006; Berghorst et al., 2013). It is also unclear the extent to which either effect persists in the aftermath of the experience of stress. Nevertheless, if a stress-driven disruption of decision-making processes is present, it is likely to affect performance. The few comparisons between pre- and post-pandemic periods available in the literature have yielded mixed evidence. For instance, Pilotti et al. (2023a) found that performance after the pandemic improved relative to that of the pandemic. Specifically, a return to fully face-to-face classes was accompanied by performance higher or equivalent to that of pre-pandemic levels. Zheng and Zheng (2023), instead, found a decline.

According to Gonzalez et al. (2020), the stay-home restrictions of the pandemic have altered the way students study, from a discontinuous approach shaped by looming deadlines and often punctuated by cramming to a more distributed approach. Gonzalez et al. argued that this habit shift explains the findings of students’ enhanced academic performance during the pandemic relative to the pre-pandemic period (Gonzalez et al., 2020; Iglesias-Pradas et al., 2021). Yet, evidence exists that this habit shift has been challenged by a return to campus life after the pandemic (Pilotti et al., 2023c). Assuming that study habit reversals to the pre-pandemic period have occurred, it is unclear whether they may be accompanied by changes in the accuracy and confidence of performance estimations.

The present study adds to the existing body of knowledge regarding performance predictions by examining an understudied population of learners who are under intense pressure to succeed. Since before the pandemic, such pressure has come from the neo-liberal economic plan of their country (Saudi Arabia), which heavily relies on college-educated youth to quickly transform the economy from a fossil-fuel based to one that is knowledge-driven. As a result of the plan, considerable financial resources have been invested in learners from elementary school to college and beyond (Le Ha and Barnawi, 2015; Nurunnabi, 2017; Tayan, 2017), reshaping the entire educational system to meet Western standards. An accidental byproduct of the education and corporate worlds’ attempts to quickly develop a suitable workforce is to reward “good grades” with generous scholarships and professional opportunities. Not surprisingly, students are struggling to find a balance between grade orientation (i.e., obtaining tangible signs of success, such as grades and a degree) and learning orientation (i.e., acquiring knowledge that ensures future professional success; Pilotti et al., 2023b). Yet, grade orientation, although unavoidable, is associated with negative emotional states, such as anxiety (Pilotti et al., 2023b). Thus, within this population, the aftermath of stress from the pandemic (AlHadi and Alhuwaydi, 2023) may add to the underlying stress resulting from the persistent intense pressure to succeed. According to the Yerkes-Dodson law, excessive arousal harms human functioning (Yerkes and Dodson, 1908). In this context, learners’ ability to predict their performance (as measured by grades) becomes critical to their ability to cope with the academic demands of the post-pandemic world with its face-to-face classes and added time-management challenges (e.g., commuting times, increased socialization, and participation in extra-curricular on-campus activities). Accurate predictions of performance are related to academic attainment as they reflect learners’ effective self-regulation (Foster et al., 2016; Knight et al., 2022). As noted earlier, self-regulation refers to strategies that learners use to monitor and control their cognitive performance before, during, and after studying (Li et al., 2018).

In the present field study, students predict their grades on the final exam of a class in which they are enrolled after having returned to on-campus instruction (i.e., in the aftermath of a stressful event). They also indicate their confidence in the predictions made. Predictions and subjective confidence ratings occur at two points in time (i.e., before and after the exam) to determine the extent to which varied outcome uncertainty can shape students’ predictions and confidence. A set of contrasting hypotheses are tested:

*H*1: If the illusion-of-knowing bias is operative, poor or deficient performers will overestimate their exam grades and will be reasonably confident in their predictions.

*H*2: If the optimism bias is operative, poor or deficient performers will overestimate their exam grades but will do so with little or no confidence in their predictions.

*H*3: Students will be unlikely to rely on the pessimistic-bracing bias because knowledge of the outcomes of the exam will not be available for weeks (i.e., objective information is distant).

*H*4: If good performers are not affected by any of these biases, they will either be accurate or slightly underestimate their likely performance as well as be confident in their estimates.

*H*5: The extent to which students can process objective information from the exam to calibrate their predictions and subjective confidence will be indexed by changes in the estimations made and confidence expressed before and after the exam. This hypothesis rests on the assumption that people may depart from either optimism or pessimism as a response to information bearing on the accuracy of their predictions (e.g., the concrete experience of having answered exam questions; Carroll et al., 2006; Saenz et al., 2017; Knight et al., 2022).

In the field of education, various empirical studies have been carried out on discrepancies between students’ grade predictions and actual grades. However, to our knowledge, studies have focused on specific majors, such as those under the umbrella of Science, Technology, Engineering, and Math (STEM) (Knight et al., 2022; Romero-Abrio and Hurtado-Bermúdez, 2023), or have overlooked the matter. Interestingly, machine learning algorithms find it easier to predict grades in STEM than non-STEM subjects (Denes, 2023). The reason is that there are qualitative differences in grading practices (King, 2015; Witteveen and Attewell, 2020; Tomkin and West, 2022). Grading practices in non-STEM subjects are more subjective, introducing noise into the input and output features of the algorithms (Denes, 2023). Thus, in our study, we differentiate students based on their major: STEM and non-STEM.

*H*6: If indeed STEM and non-STEM students have been exposed to different grading practices (Witteveen and Attewell, 2020; Tomkin and West, 2022), STEM students’ experience with less subjective grading may make grade estimation more accurate and confident than that of non-STEM students.

## 2 Methods

### 2.1 Participants

Participants were a convenience sample of 799 students. They were enrolled in a course of the general education curriculum devoted to research writing after returning to on-campus instruction. At the time the study was conducted, all instruction was face-to-face. The selection of a general education course required during the first year of enrollment ensured that the sample would represent freshmen in all academic majors. In this sample, 47.81% were students enrolled in STEM programs (e.g., computer science, engineering, or architecture) whereas 52.19% were students enrolled in non-STEM programs (e.g., law, business, and interior design). Their ages ranged from 18 to 25 years. All students were Arabic-English bilingual speakers for whom English competency was confirmed through standardized tests before enrollment. At the selected institution, English is the primary means of instruction for a curriculum of US import fully administered on campus after the pandemic. All students are commuters as there are no residential halls on campus.

### 2.2 Procedure and materials

Students were given 2 h to complete a final exam. The exam served as a summative assessment measure. The final exam for students’ grade predictions was chosen based on course evaluation ratings and spontaneous comments collected by instructors inside and outside the classroom. Reasons included its (a) relevance (i.e., students viewed the exam as key to their overall performance in the course); (b) outcome uncertainty (i.e., students described the exam as comprehensive and challenging, thereby yielding uncertain results); and (c) outcome distance (i.e., the actual results of the exam were not immediately known). Although the exact format of the final exams varied by instructor, consistency was high across the sections of the course included in the present study. Final exams consisted of a mixture of short-answer questions and multiple-choice questions that engaged four of the six levels of the Bloom taxonomy (Krathwohl, 2002; Nkhoma et al., 2017): understanding, application, analysis, and evaluation.

The first and last sheets of the final exam contained estimation and confidence-rating questions. Before completing the exam, students were asked to estimate their grade on the exam and indicate the confidence with which their estimation was made on a scale from “not confident at all” (0) to “very confident” (4). After the examination was completed, the same questions were asked again. At each time, they were reminded that estimations needed to be realistic rather than aspirational.

After responses and grades were entered into a spreadsheet, identifying information was deleted and random number codes were used to uniquely identify each participant’s set of responses. The study was approved by the Deanship of Research as conforming to the standards for educational research of the Office for Human Research Protections of the US Department of Health and Human Services.

## 3 Results

SPSS was utilized for data analysis. The participants’ exam scores (range 0–100) were organized into three categories: good performance (100–80%: from A+ to B), poor performance (79–66%: from C+ to D+), and deficient performance (65–0%: from D to F). At the selected institution, a passing grade corresponds to a D+. Grade categories were chosen to align with institutional standards.

Tables 1–3 report descriptive statistics, including grades, grade estimates, and subjective confidence ratings. Inferential statistics were used for hypothesis testing (H1–H6). They involved between-subjects ANOVAs followed by *post hoc* (Bonferroni) tests to identify differences. All results of inferential statistics were considered significant at the 0.05 level.

**Table 1:** Mean (M) exam grades and standard deviation (SD) as a function of performance level (good, poor, and deficient) and major (STEM and non-STEM).

Performance level	Good		Poor		Deficient	
	*M*	*SD*	*M*	*SD*	*M*	*SD*
STEM	90.73%	6.52	72.64%	3.67	50.66%	12.09
*n*	160		93		129	
Non-STEM	90.24%	6.59	73.29%	3.79	48.51%	13.15
*n*	176		96		145	

*n* refers to the number of students at each performance level.

**Table 2:** Mean (M) accuracy and standard deviation (SD) before the exam as a function of performance level (good, poor, and deficient) and major (STEM and non-STEM).

Performance level	Good		Poor		Deficient	
	*M*	*SD*	*M*	*SD*	*M*	*SD*
Accuracy before the exam						
STEM	−1.08%	11.21	+13.36%	11.05	+30.97%	18.93
Non-STEM	−0.17%	11.68	+10.69%	13.63	+28.03%	20.74
Combined	−0.63%		+12.03%		+29.50%	
Confidence before the exam						
STEM	2.47	0.92	2.17	1.05	2.08	1.13
Non-STEM	2.50	1.01	2.11	0.92	2.01	1.14
Combined	2.49		2.14		2.05	

**Table 3:** Mean (M) accuracy and standard deviation (SD) after the exam as a function of performance level (good, poor, and deficient) and major (STEM and non-STEM).

Performance level	Good		Poor		Deficient	
	*M*	*SD*	*M*	*SD*	*M*	*SD*
Accuracy after the exam						
STEM	−4.78%	12.15	+5.85%	14.28	+17.30%	18.20
Non-STEM	−4.51%	12.22	+5.06%	17.19	+18.05%	20.92
Combined	−4.65%		+5.46		+17.68	
Confidence after the exam						
STEM	2.19	1.11	1.86	1.07	1.56	1.10
Non-STEM	2.19	1.02	1.96	1.16	1.61	1.10
Combined	2.19		1.91		1.59	

A 3 (performance level) × 2 (major) between-subjects ANOVA yielded a main effect of performance level [*F*(2, 793) = 1649.62, *MSE* = 73.33, *p* < 0.001, *η_p_*^2^ = 0.806], but no other effects (*Fs ≤* 1.51). Exam performance increased from deficient to good for both STEM and non-STEM students (see Table 1).

For each student, accuracy scores were computed by subtracting the grade received from the grade predicted either before or after the exam. A positive score indicated an inflated (optimistic) estimation, a negative score signified a deflated (pessimistic) estimation, and a score of 0 implied an accurate prediction. Tables 2, 3 contain the descriptive statistics for the accuracy and confidence ratings. Statistical analyses were organized by the questions they answered.

### 3.1 Did accuracy and confidence vary with performance?

A 3 (performance level) × 2 (major) between-subjects ANOVA was carried out on the accuracy scores before the exam when uncertainty regarding the outcomes was maximal. In this analysis, the only significant effect was that of performance level [*F*(2, 793) = 300.12, *MSE* = 227.67, *p* < 0.001, *η_p_*^2^ = 0.431], indicating that predictions increased in accuracy with performance (other *F*s ≤ 2.04). Estimates were overall rather realistic for good performers. Poor or deficient performers inflated their predictions. This pattern fits both the illusion-of-knowing bias and the optimism bias.

A 3 (performance level) × 2 (major) between-subjects ANOVA was also carried out on the confidence scores before the exam to determine which phenomenon could best fit the data. The only significant effect was that of performance level [*F*(2, 793) = 15.41, *MSE* = 1.07, *p* < 0.001, *η_p_*^2^ = 0.037; other *F*s ≤ 1]. Contrary to the accuracy scores, confidence decreased with performance. Namely, poor or deficient performers were less confident in their predictions than good performers who yielded more realistic predictions. This pattern of effects supports the optimism bias account. Thus, H2 was supported by the accuracy and subjective confidence data. H4, which predicted that good performers would yield realistic estimates and be confident in such estimates, was supported. In contrast, H1 (i.e., illusion-of-knowing prediction) and H3 (i.e., pessimistic-bracing bias prediction) were not supported. Also not supported was the prediction that STEM students’ experience with less subjective grading would make grade estimation more accurate and confident than that of non-STEM students (H6).

### 3.2 Did reality tempter accuracy and confidence?

Evidence that deficient and poor performers were able to moderate their estimates by taking into consideration the information provided by the exam would further support the optimism bias account. To examine this issue, a 3 (performance level) × 2 (major) × 2 (time) mixed-factorial ANOVA was carried out on the accuracy scores before and after the exam. The analysis yielded a main effect of time [*F*(1, 793) = 267.62, *MSE* = 78.58, *p* < 0.001, *η_p_*^2^ = 0.252], indicating that estimates changed from before to after the exam. There was also a main effect of performance level [*F*(2, 793) = 256.39, *MSE* = 403.58, *p* < 0.001, *η_p_*^2^ = 0.393], underscoring that the predictions made by students with differing levels of performance varied. An interaction between performance level and time of the estimate [*F*(2, 793) = 29.59, *MSE* = 78.58, *p* < 0.001, *η_p_*^2^ = 0.069] indicated an uneven pattern. Deficient or poor performers tempered their optimistic estimates after having encountered the test, thereby reflecting the integration of reality into their expectations. Specifically, students with deficient performance reduced their estimates from +29.50% to +17.68% (a drop of 11.82%). Similarly, students with poor performance reduced their estimates from +12.03% to +5.46% (a drop of 6.57%). Instead, good performers became more cautious, thereby underestimating performance (from −0.63% to −4.65%). No other effects reached significance (*F*s ≤ 3.25, *ns*).

A 3 (performance level) × 2 (major) × 2 (time) mixed-factorial ANOVA was also carried out on the confidence scores before and after the exam. In this analysis, a main effect of time [*F*(1, 793) = 80.76, *MSE* = 0.51, *p* < 0.001, *η_p_*^2^ = 0.092] indicated that students’ subjective confidence declined after the exam. A main effect of performance level [*F*(2, 793) = 24.23, *MSE* = 1.75, *p* < 0.001, *η_p_*^2^ = 0.058] underscored that confidence varied with performance. An interaction between performance level and time [*F*(2, 793) = 3.18, *MSE* = 0.51, *p* = 0.042, *η_p_*^2^ = 0.008] indicated that the declines in confidence after the exam differed among performance level groups. Deficient or poor performers reduced their confidence in the estimates made after the exam by 0.49 and 0.23, respectively. Good performers’ confidence declines were between the two groups (0.30). No other effects reached significance (*F*s ≤ 1.30, *ns*).

These data support the optimism bias account (H2). Evidence that poor or deficient performers integrated the knowledge gathered from the exam into their predictions and confidence ratings suggests that reality punctured their optimism. H5 was supported. No evidence was found that STEM and non-STEM students’ grade estimation would differ in accuracy and confidence, thereby failing again to find support for H6.

## 4 Discussion

Our results can be summarized in three points. First, grade estimation and confidence ratings supported H2. Indeed, poor or deficient performers overestimated their expected exam grades, but their optimistic predictions were tempered by their weak confidence in the predictions made. Relative to such students, good performers were reasonably accurate in their grade predictions (at least before the exam) and more confident in their predictions, thereby supporting H4. Miller and Geraci (2011a) also found that low-performing students make grade predictions with less confidence than high-performing students. Svanum and Bigatti (2006) and Pilotti et al. (2023c) argued that for students whose performance is less than desirable, optimistic predictions may be the expression of wishful thinking.

Second, all students were able to revise their predictions based on the information gathered from completing the exam, thereby supporting H5. The optimistic estimates of deficient performers shrank twice as much as those of poor performers. The estimates of good performers became pessimistic. After the exam, confidence in the estimates made also declined for all learners. However, the steepest decline was yielded by deficient performers. These findings, which replicate those of Pilotti et al. (2021, 2023c), suggest that learners’ metacognitive experiences before and after the exam are different. Before the exam, the key sources of estimates are study activities and scant information about the format and content of the exam gathered from the instructor and past students. After the exam, the key source is the experience of taking the exam. Pre-exam judgments regarding upcoming performance might be more susceptible to the effects of desires than post-exam judgments. The reason is that the latter can be based on less uncertain information.

Findings of revised performance predictions following the concrete experience of the final exam contradict the assumption that students with poor or deficient performance are unaware of their shortcomings, which is implied by the illusion-of-knowing phenomenon (Williams, 2004; Ehrlinger et al., 2008; Miller and Geraci, 2011a; Serra and DeMarree, 2016). Saenz et al. (2017) also reported that, if students are warned against relying on desired grades, they can improve the accuracy of their predictions. In their study, they found that low performers are the primary beneficiaries of improved calibration following this motivation debiasing intervention. Thus, even poor or deficient performers are not blind to (a) the factual information offered by the direct experience of an exam (as in our study) or (b) instructions focusing their attention on their actual (rather than aspirational) abilities to complete successfully a particular exam (as per Saenz et al., 2017). Motivation debiasing instructions may be successful because they direct students’ attention to objective performance information (e.g., prior performance and current knowledge) as much as direct experience with an exam does.

Third, grade estimation and confidence of STEM and non-STEM students did not differ. H6 was not supported. One of the reasons might be that the students selected for the study were at the start of their academic journey. They were freshmen and sophomores whose enrollment mostly entailed general education courses rather than major courses. As such, experiences with the grading practices of STEM and non-STEM fields in academia were too meager to yield a noticeable difference.

Serra and DeMarree (2016) and Saenz et al. (2017) suggested that students have a difficult time distinguishing desired grades from expected grades. Do learners base their predictions of academic performance on how they wish to perform and disregard how they expect to perform? To answer this question, in pilot work, a subset of students were asked before the final exam to indicate their desired grades (i.e., “What grade do you realistically wish to earn on the final exam in this course?”). Based on students’ cumulative grades, 20 good performers and 20 deficient performers were questioned. We found that all students consistently reported desired grades in the range of an A+ even though students were reminded to express realistic desires. Their estimated final exam grades were consistently lower than their desired grades. Thus, students can distinguish between desired and likely outcomes. It is possible though that students use desired grades as a psychological anchor (i.e., mental starting point) for grade predictions (Scheck et al., 2004; England and Serra, 2012). Poor and deficient learners may not carefully consider additional information (e.g., performance on practice tests) to adjust their initial estimates. By doing so, they temporarily preserve their self-concept and mitigate the dreadful information they will be likely to face. Good performers may also start with an anchor based on their desires but their self-concept will not be jeopardized by a self-examination of their knowledge and skills. Thus, they can mentally adjust their estimates before arriving at a value to be confidently reported. Because a more thorough evaluation process is likely to be carried out by good performers, poor and deficient performers are also less sure of their predictions (Miller and Geraci, 2011a; Händel and Fritzsche, 2016). Yet, when confronted with the inescapable reality of the exam, even poor and deficient performers are not blind to it. On the contrary, they adjust their estimates much more than good performers, but their estimates remain optimistic, thereby suggesting that they attempt to protect their self-concept for a little longer.

Our results inform metacognitive interventions for at-risk students. It is important to note that for learners, metacognition is a window into their thought processes (Dunlosky and Metcalfe, 2009; Briñol and DeMarree, 2012), which plays a role in determining how students learn and perform. Learners rely on (a) metacognitive knowledge to determine how to study, (b) metacognitive monitoring to evaluate how well they know the materials they are currently studying, and (c) metacognitive control and monitoring to decide to either continue or stop studying (Dunlosky and Metcalfe, 2009). If any of these aspects of metacognition malfunctions, students’ learning may be impaired (Serra and Metcalfe, 2009).

Interventions to temper students’ deviations from realistic self-assessment mostly focus on metacognitive knowledge and monitoring. For instance, Callender et al. (2016) suggest instructing students on the consequences of unrealistic estimation, offering concrete feedback about their performance, and even considering incentives for accurate calibration. Saenz et al. (2019) report that reviews, incentives, and students’ independent reflections on their performance estimations do not improve prediction accuracy. Effective methods include feedback about students’ performance and related grade predictions immediately after tests as well as lectures about relying on actual performance and familiarity with study materials to inform grade predictions and related studying. Al Kuhayli et al. (2019) agree that an effective shield against biased estimations, especially for low performers, is practice with estimation followed by feedback. Because Buckelew et al. (2013) find that at-risk students not only overestimate their grades but also attribute the grades attained to external sources, interventions intended to alter causal attribution habits may also be useful. Additional interventions may focus on altering other learners’ characteristics, such as self-efficacy beliefs (i.e., confidence in one’s abilities), as a vehicle to improve academic performance and, with it, metacognition. As noted by Golke et al. (2022), high self-efficacy may be related to overestimations. Thus, one of the goals of an effective intervention may be to modulate students’ self-efficacy to achieve a middle ground between deficient confidence and excessive confidence.

The limitations of the present study include the participants who were by and large freshmen. If the sample were to include juniors and seniors, differences between STEM and non-STEM majors might have been detected. The study was conducted in real classrooms rather than in labs. Thus, potentially consequential individual differences were not controlled (Kroll and Ford, 1992; Kelemen et al., 2000; Händel et al., 2020; Golke et al., 2022). Furthermore, in the current study, participants did not receive performance feedback for any of the class activities preceding the final exam. If they were given practice with estimation and feedback, one might expect more accurate predictions and higher confidence, especially for poor or deficient performers (Al Kuhayli et al., 2019). Qualitative information regarding students’ explanations for their performance, estimates, and confidence ratings might also be useful (see Gutierrez de Blume and Montoya Londoño, 2021). Furthermore, no examination of their state of mind, including the experience of stress, was performed. As such, it is unclear how their current affective state might have shaped not only estimates of grades but also performance. Lastly, as suggested by Tang et al. (2022), longitudinal research conducted on large-scale samples might be advisable if strategies to improve students’ learning outcomes are sought.

## 5 Conclusion

The pandemic may have affected Saudi Arabian students’ lives and altered their study habits and academic performance as it has done for many other college students around the world (Algahtani et al., 2021; Madrigal and Blevins, 2022). Yet, for the selected sample of Saudi Arabian students, it has not visibly changed the metacognitive pattern of performance estimation that was uncovered before the pandemic. In the post-pandemic period, these students face a top-down intense pressure to succeed, which has preceded the pandemic, along with the common situational stress of college life (examinations, deadlines, etc.). Even under these convergent forces, their estimation patterns are not different from those of students in the Western world (Miller and Geraci, 2011a; Hamann et al., 2020) collected before the pandemic. How can one explain these findings? It is possible that in this sample of students, converging pressure forces do not lead to stress at a level at which it can disrupt grade estimation processes in working memory. Alternatively, students may have adapted to such forces, thereby neutralizing their impact by developing adaptive strategies in response to adverse conditions (Ellis and Del Giudice, 2019). Whether adaptation to stress may be the result of their habitual outlook on life, including how they appraise, interpret, and react to adverse events (Ong et al., 2006), is to be determined.

## Data availability statement

The raw data supporting the conclusions of this article will be made available by the authors without undue reservation.

## Ethics statement

The research, which involved human subjects, was approved by the Deanship of Research of Prince Mohammad Bin Fahd University in accordance with its Ethics Committee Board’s practices. The study was conducted in accordance with local legislation and institutional requirements. The participants provided their written informed consent to participate in this study. The study was conducted in accordance with the local legislation and institutional requirements.

## Author contributions

MP: Conceptualization, Formal analysis, Investigation, Methodology, Project administration, Writing – original draft, Writing – review & editing. KE: Conceptualization, Formal analysis, Investigation, Methodology, Writing – original draft, Writing – review & editing. AW: Conceptualization, Formal analysis, Investigation, Methodology, Writing – original draft, Writing – review & editing.
